# Contingency and determinism in the evolution of bird song sound frequency

**DOI:** 10.1038/s41598-021-90775-6

**Published:** 2021-06-02

**Authors:** Jakob I. Friis, Torben Dabelsteen, Gonçalo C. Cardoso

**Affiliations:** 1 Behavioural Ecology Group, Department of Biology, University of Copenhagen, Copenhagen Ø, Denmark; 2 CIBIO/InBIO, Centro de Investigação em Biodiversidade e Recursos Genéticos, Universidade do Porto, Vairão, Portugal

**Keywords:** Animal behaviour, Evolution, Sexual selection

## Abstract

Sexual signals are archetypes of contingent evolution: hyper-diverse across species, often evolving fast and in unpredictable directions. It is unclear to which extent their evolutionary unpredictability weakens deterministic evolution, or takes place bounded by deterministic patterns of trait evolution. We compared the evolution of sound frequency in sexual signals (advertisement songs) and non-sexual social signals (calls) across > 500 genera of the crown songbird families. Contrary to the acoustic adaptation hypothesis, we found no evidence that forest species used lower sound frequencies in songs or calls. Consistent with contingent evolution in song, we found lower phylogenetic signal for the sound frequency of songs than calls, which suggests faster and less predictable evolution, and found unpredictable direction of evolution in lineages with longer songs, which presumably experience stronger sexual selection on song. Nonetheless, the most important deterministic pattern of sound frequency evolution—its negative association with body size—was stronger in songs than calls. This can be explained by songs being longer-range signals than most calls, and thus using sound frequencies that animals of a given size produce best at high amplitude. Results indicate that sexual selection can increase aspects of evolutionary contingency while strengthening, rather than weakening, deterministic patterns of evolution.

## Introduction

Determinism in evolution refers to lineages under similar selective pressures converging into similar phenotypes, while evolutionary contingency can lead to those lineages diverging phenotypically, for example due to the random appearance and loss of genetic variation, and to stochasticity in evolutionary trajectories^1^. Evolutionary contingency refers broadly to unpredictability in evolutionary trajectories, even under identical starting points and selective pressures^2^. It is expected that traits undergoing evolutionary contingency change frequently, because multiple selective optima may exist without the selective landscape needing to change, and that the phenotypes of closely-related lineages diverge easily, resulting in low phylogenetic signal. A related meaning of the term evolutionary contingency is that of historical contingency, which refers to clade-specific constraints or differences in the response to selection^3,4^, and which causes high rather than low phylogenetic signal. For example, the evolution of species distinctiveness in woodpecker drums follows clade-specific rules and, accordingly, the acoustic structure of drums has high phylogenetic signal^5^. Here we do not use the term contingency in the sense of historical contingency, but under the more general definition of unpredictable and changing evolutionary trajectories.

We hypothesize that some types of selection, namely sexual selection, may simultaneously increase contingent aspects of evolution (e.g., fast rates of change in unpredictable directions), and also increase the strength of deterministic boundaries within which those contingent effects take place. In such a scenario, increased contingency might not weaken, and could even strengthen, deterministic patterns of trait evolution. Sexually-selected signals and ornaments are notable examples of contingency in phenotypic evolution because they are hyper-diverse across species, they are frequently lost and re-gained evolutionarily^6^, and they can evolve rapidly, such that related lineages often differ in sexual signals while being phenotypically very similar otherwise (e.g.^7–9^). For example, avian lineages under stronger sexual selection often have faster rates of phenotypic evolution on song^10,11^ or plumage colors^12–14^, but they do not necessarily evolve towards similar phenotypes^10,11^. In the case of bird song, behavioral research has even found that diametrically opposed sound frequencies can fulfil identical functions: in some species lower sound frequencies are preferred in courtship or are used to signal aggression, while in other species it is higher frequencies that fulfil those functions (reviewed in^15^).

Despite sexual signals being archetypes of contingent evolution, they are also subject to deterministic selective pressures^12^. Continuing with the case of sound frequency, well-known deterministic effects on its evolution are due to body size and habitat type. Acoustic theory predicts a negative linear relation between log-body mass and log-frequency of acoustic signals^16,17^ because, all else being equal, larger vocal organs and vocal tracts produce and resonate low frequencies more efficiently, and are better at coupling low frequencies to the surrounding medium^18^. To a lesser extent, forested habitats can also cause evolution of low sound frequencies^19,20^, mostly because foliage scatters and dampens the transmission of high frequencies^21–23^.

It is unclear to which extent the contingency associated with sexual selection weakens deterministic evolution (i.e., weakening cross-species associations between properties of sexual signals and their morphological or ecological predictors), as opposed to contingent effects taking place bounded by deterministic patterns of trait evolution^12^. To address this, here we compared deterministic patterns of sound frequency evolution between bird songs, which are an important type of acoustic sexual signal, and, as a control, contact calls, which are acoustic signals that include more general, non-sexually-selected social functions. We tested whether sound frequency in songs shows signs of contingent evolution, and whether or not its evolution became less deterministic than in calls.

The advertisement songs of songbirds are sexual signals typically used in mate attraction and/or competition with rivals^24^, and differences in their sound frequency are often implicated in female preferences or other sexual functions (reviewed in^15^). While songs are often sung solely or mostly by adult males^25^, other types of vocalizations, such as contact calls, are used by all individuals and have mostly non-sexual social functions^26^. We used the crown families of songbirds (i.e., parvorder Passerida^27,28^), which comprises ca. 77% and 75% of all songbird species and genera, respectively^29^, because it is generally straightforward to identify advertisement song and one main type of contact call in these species. We used descriptions in the literature to identify the main call type of each species or, when such descriptions were lacking, we used the most commonly recorded call (excluding alarm calls) as the main, most representative call type of the species. We restricted our study to the Passerida because in clades splitting from more basal nodes in the songbird phylogeny (e.g., Corvoidea or Meliphagoidea) it is common that song is less elaborate or less used, and that birds have various different call types^30,31^, making songs and contact calls not as easily distinguishable and compared.

**Figure 1:**
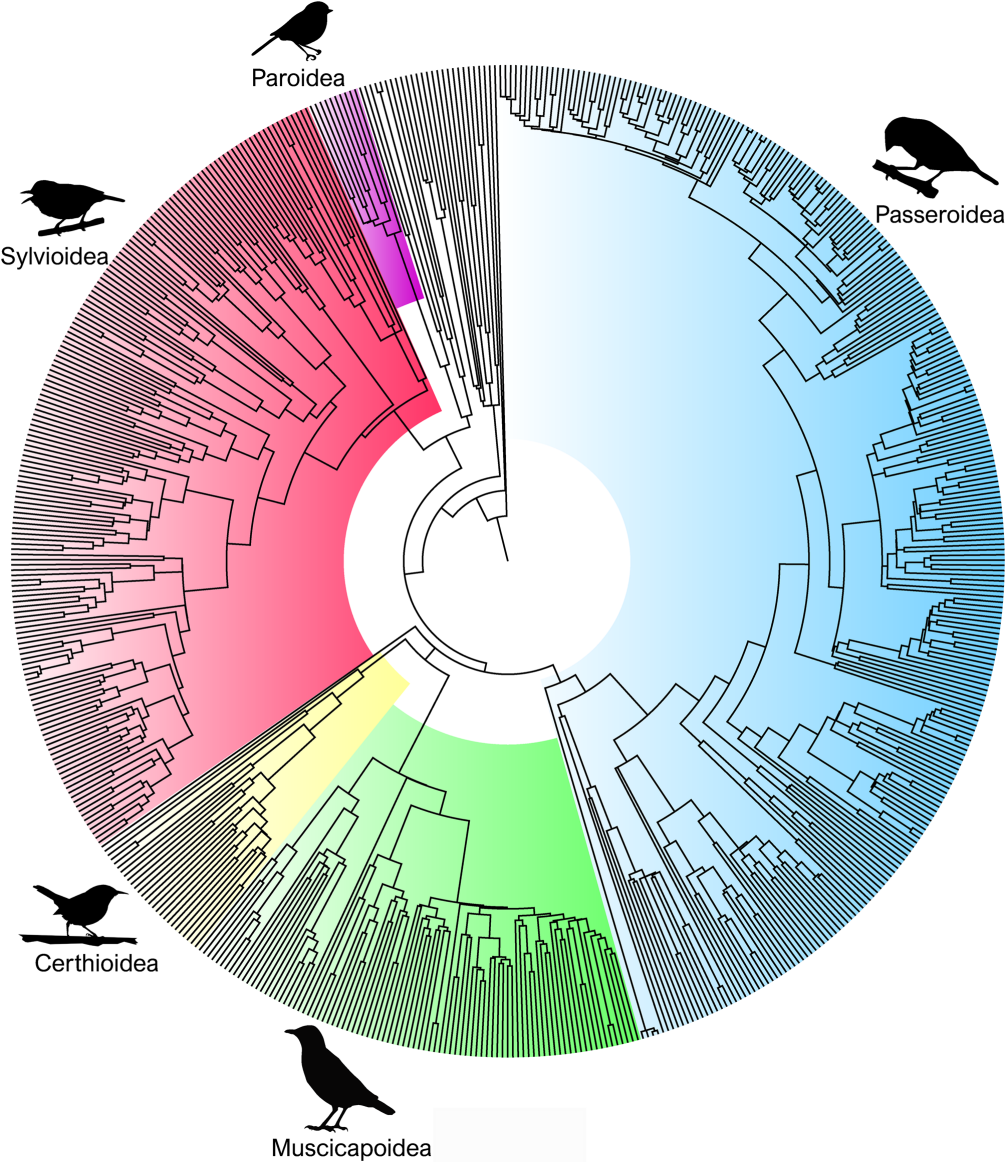
Phylogenetic tree, colored by superfamily. Example of a phylogenetic tree including all studied genera, out of the 1000 trees used to account for shared ancestry. All analyses used Akaike’s information criterion-model averaging across phylogenetic generalized least squares model outputs from these 1000 trees. See the “Supporting information”, Appendix S1, for the assignment of superfamilies. Bird silhouettes, here and in Figs. 2 and 3, are by Ferron Sayol and Matt Wilkins, from phylopics.org, and are in the public domain (creative commons license CC0 1.0).

We gathered and measured song and call recordings in more than 500 genera of Passerida (Fig. 1), to compare the evolution of sound frequency in songs and calls. Since sexually-selected signals typically evolve in a highly contingent manner, with phenotypic changes in unpredictable or opposed directions^6^, we expected that the sound frequency of songs should be more evolutionary labile and thus show weaker phylogenetic signal^32,33^ than that of calls. To ask if species experiencing stronger sexual selection on song tend to evolve sound frequency in one particular direction or in unpredictable directions, we also measured song duration as a proxy for song elaboration and, therefore, for the strength of sexual selection specifically on song. We used song duration because it is an aspect of song motor performance, and because song duration correlates with syllable diversity and repertoire size across species (e.g.,^34,35^). We did not use more general indexes for the strength of sexual selection, such as color ornamentation or sexual dichromatism, because species differences in ornamentation are generally not associated with song elaboration (reviewed in^36,37^) and, therefore, these indexes may bear little relation to the strength of sexual selection acting specifically on song. Finally, regarding deterministic evolution, and since body size and habitat type are the two best understood predictors of sound frequency in acoustic signals^16,17,19^, we evaluated the strength of deterministic evolutionary patterns by the strength of the cross-species association between sound frequency and either body size or habitat type.

## Results

Body mass (log_10_g) and habitat type (proportion of time foraging in forests) had high phylogenetic signal (*λ* = 0.89 and 0.75, respectively), indicating a good fit between species similarity and phylogenetic relatedness, while song duration (log_1016_s) had only moderate phylogenetic signal (*λ* = 0.45), indicating labile evolution with more divergences and convergences across the phylogeny. The phylogenetic signal of song frequency was of intermediate value (*λ* = 0.59), and lower than that of call frequency (*λ* = 0.79), indicating more labile evolution of song than call frequencies along the phylogeny.

**Figure 2:**
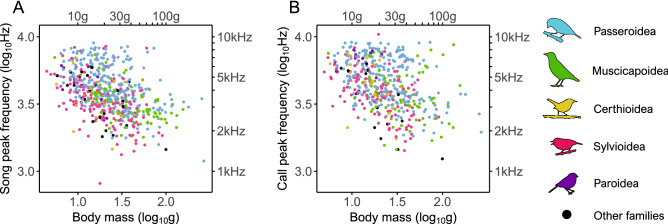
Relation of peak frequency and body mass across species. (**A**, **B**) Scatterplots of peak frequency and body mass for (**A**) songs or (**B**) calls. Species are colored by superfamily.

The sound frequency of songs and calls were negatively related to body size (Fig. 2), but not significantly related to habitat type (phylogenetic generalized least squares [PGLS] for song frequency: partial *β*_*st*_ of body size = − 0.51, P < 0.001, partial *β*_*st*_ of habitat = − 0.02, P = 0.51, N = 591 spp., model *λ* = 0.31; PGLS for call frequency: partial *β*_*st*_ of body size = − 0.38, P < 0.001, partial *β*_*st*_ of habitat = − 0.02, P = 0.58, N = 505 spp., model *λ* = 0.65). These results, from multiple PGLS regression, are similar to results using simple PGLS regression models of peak frequency on body size alone (songs: *β*_*st*_ = − 0.51, P < 0.001, model *λ* = 0.31; calls: *β*_*st*_ = − 0.38, P < 0.001, model *λ* = 0.65) or of peak frequency on the proportion of time in forested habitat (songs: *β*_*st*_ = − 0.00, P = 0.96, model *λ* = 0.59; calls: *β*_*st*_ = 0.03, P = 0.48, model *λ* = 0.78). Despite the more labile evolution of sound frequency in songs than calls, we found that the association with body size was significantly stronger for songs (*β*_*st*_ = − 0.51, Fig. 2A) than calls (*β*_*st*_ = − 0.38, Fig. 2B; t-test: Z = − 2.05, two-tailed P = 0.04).

**Figure 3:**
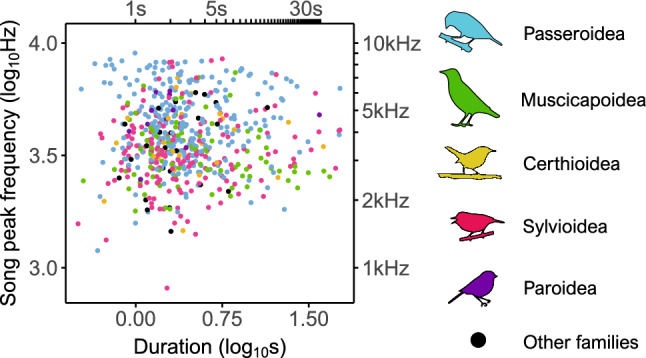
Relation of song peak frequency and song duration across species. Scatterplot of song peak frequency on song duration across species. Species are colored by superfamily.

Song duration, used here as a proxy for the strength of sexual selection on song, was not related to the sound frequency of songs (PGLS: partial *β*_*st*_ of song duration = 0.03, P = 0.44, partial *β*_*st*_ of body size = − 0.52 P < 0.001, and partial *β*_*st*_ of habitat = − 0.03 P = 0.49, N = 591 spp., model *λ* = 0.31; Fig. 3). Inspection of Fig. 3 also shows that the variance of song frequency remains identical across species with short or long songs. To test this, we used residual frequencies from the PGLS regression of sound frequency on body size and habitat, to control their effects on sound frequency, and regressed the absolute values of those residual frequencies on song duration. We found that absolute values of residual frequency were also not related to song duration (PGLS: *β*_*st*_ = 0.01, P = 0.37, N = 591 spp., model *λ* = 0.96), indicating that song duration does not predict the extent of deviations from the frequencies predicted by body size.

## Discussion

A degree of contingency is expected in the evolutionary trajectories of sexually-selected traits, with frequent changes in different directions, because of the unstable nature of sexual selection and because of the multiple signals and ornamental phenotypes that it can target^6^. In agreement with this expectation, we found a lower phylogenetic signal for the sound frequency of songs than calls, indicating more labile evolution in songs, with more frequent divergence of closely-related species and convergence of distantly-related species. Since sound frequency of songs and calls is distributed over the same frequency range across species (Fig. 2), the lower phylogenetic signal of sound frequency in songs also implies that, on average, it evolved at a faster rate than in calls. Our result thus supports previous work concluding that sexual selection causes fast and diversifying phenotypic evolution (e.g.,^7–9^), including work on bird song^10,11^ and ornamentation^12–14^.

Importantly, despite the more labile evolution of sound frequency in songs than calls, we found a stronger allometry of sound frequency for songs than for calls. The negative-slope allometry of sound frequency across species that we found, whereby larger species use lower frequencies, was documented earlier in many taxa^38–40^, including for bird songs^41–44^ and calls^45^. But ours is the first work analyzing songs and calls in a sufficiently large number of species to compare the strength of their allometries. The stronger allometry of sound frequency in song can be explained by songs being, on average, longer-range signals than contact calls^46^, and needing to transmit across long distances to attract mates or repel rivals. Therefore, songs should be more strongly selected to use sound frequencies that, for a given body size, animals can best produce at high amplitude. Interestingly, body size had very high phylogenetic signal (*λ* = 0.89), the highest among all traits that we studied, which makes it more remarkable that the phylogenetic signal of sound frequency was lower in songs than calls. Together, the lower phylogenetic signal and stronger allometry of sound frequency in songs than calls indicate that the greater lability of sound frequency evolution in song took place within stronger deterministic boundaries set by body size, rather than weakening those allometric boundaries.

While body size is one of the best-understood^16,17^ and strongest correlates of sound frequency across avian species^41–44,47^, it has long been known that differences in the acoustic properties between forested and non-forested habitats can also influence the evolution of sound frequency in signals, as per the acoustic adaptation hypothesis^21–23^ (AAH). Nonetheless, our results showed a striking absence of an association between habitat type and the sound frequency of either songs or calls. This may not be unexpected at the broad taxonomic scale of our study, since reviews of the AAH showed that effect sizes of habitat type on avian sound frequencies are generally small and variable across taxonomic groups^19,20^, so that the effects of the AAH may not be as large or widespread as suggested by early studies^21,42,48^. Similarly to our results, other large-scale tests of the AAH for the sound frequency of songs, across Neotropical Parrots^47^ and Passeriformes^44^ also did not find a significant effect of vegetation density. These studies used different proxies for habitat type (vegetation indexes from satellite imagery) than ours (time foraging from forest canopy to understory) to arrive at identical results, and yet another comparative study, across the large suboscine radiation of ovenbirds (family Furnariidae), even reported an association of forested habitat with higher song minimum frequency, which goes in the opposite direction of the AAH^49^. Together, these results indicate that, while the AAH may explain differences in sound frequency among some closely-related species (e.g.,^50^) or across finer habitat differences than those that these large-scale studies can capture (e.g., between different forest strata^51^, or across Mediterranean-type habitats^52^), forest living has little predictive power at our broad taxonomic level.

Using song duration as a proxy for the strength of sexual selection on song, we found no evidence that species with longer songs tended to evolve sound frequency in a consistent direction (either high or low frequencies) or frequencies further away from the optimum predicted by body size (in both cases effect sizes were lower than 0.1%). Inasmuch as song duration indicates the strength of sexual selection on song, these results indicate that stronger sexual selection does not create a deterministic pattern of sound frequency evolving in a consistent direction, and it also does not weaken the deterministic constraint placed by body size. Here we used song duration as a proxy to evaluate the strength of sexual selection on song because duration is an aspect of song motor performance (longer songs can place motor and ventilation challenges^53^), and song motor performance can be sexually selected^54^. In addition, song duration correlates with syllable diversity and repertoire sizes across species (e.g.,^34,35^), and sexual selection has often been found to favor long song^55^, or diverse repertoires^56^. Also, songs are generally much longer than calls (Fig. S3C,D), indicating that sexual selection often causes the evolution of longer acoustic signals. Therefore, despite sexual selection being able to target several distinct song traits^54^, when comparing across a very large number of species, it is reasonable to expect that species subject to strong sexual selection on song have, on average, evolved longer songs than species subject to weaker sexual selection on song.

Differences in the strength of sexual selection across bird species are often inferred based on traits unrelated to song, such as ornamentation, sexual dichromatism or sexual size dimorphism (SSD). We did not use proxies based on ornamentation because species differences in ornamentation are generally not associated with song elaboration (reviewed in^36,37^), and in some cases may be negatively associated^57^. Therefore, it would be misleading to use color-based indexes for inferring strength of sexual selection on song. Male-biased SSD is also an often-used proxy to infer the strength of sexual selection, especially across non-avian tetrapod species, because the evolution of SSD may be driven by male–male competition (reviewed in^58^). But many birds interact in the air, where larger male size is not necessarily advantageous for fighting or for performing sexual displays, and where smaller sizes and greater agility may be favored instead^59–65^, perhaps explaining why high sound frequencies can signal aggression or be preferred by females in several bird species (reviewed in^15^). Therefore, it is not straightforward to use male-biased SSD as a proxy for the strength of sexual selection on song, as this proxy may focus on a subset of species not representative of the various ways in which sexual selection can affect bird song. Nonetheless, previous work analyzed the sound frequency of song in relation to SSD, finding only a weak negative association with SSD across the order Passeriformes (with an effect size estimated between 1 and 3%)^44^, and this effect was not significant for the crown songbirds (Passerida; B. Kempenaers, pers. comm.). This strengthens our conclusion that sexual selection does not set a deterministic pattern of sound frequency evolution in a consistent direction.

In conclusion, our results were consistent with the expectation that sexual selection brings about evolutionary contingency (e.g.,^7–11^), here in the form of labile evolutionary trajectories, with phenotypic divergences and convergences lowering the phylogenetic signal of song frequency relative to that of call frequency or body size, and also in the form of unpredictable directions of evolution in species with longer and presumably more strongly sexually-selected songs. More importantly, we showed that this enhanced contingency did not weaken the strongest deterministic pattern of song frequency evolution, its negative-slope allometry, compared to that for calls. On the contrary, the allometry of sound frequency was significantly stronger for songs than calls, likely because long-range songs need to use frequencies that animals of a given size can produce at high amplitude. Evolutionary contingency, defined broadly as unpredictability of trajectories, contrasts conceptually with the predictability of deterministic evolution, and the two are often discussed and studied as in direct opposition, for example as when phenotypic evolution is partitioned between deterministic convergence versus among-clade divergence attributable to historical contingency (e.g.,^66,67^). But contingent and deterministic effects may interact in ways other than weakening each other^68^, for example by acting at different levels or time-scales. Our results support this more multifaceted view of the interactions between evolutionary contingency and determinism and, for the first time, indicate that sexual selection can simultaneously increase aspects of evolutionary contingency and determinism: the former at a finer time-scale, causing labile and unpredictable evolutionary trajectories, and the latter holding this labile evolution within stronger allometric boundaries.

## Methods

### Acoustic data

In summary, using the citizen-science repository Xenocanto (www.xeno-canto.org), we could obtain good-quality recordings of songs for 591 species, one species per genus, representing > 76% of Passerida genera, distributed across 62 of the 76 Passerida families (only very small families or families lacking advertisement song were not represented; Table S1). For 505 of these species, we could also obtain recordings of contact calls. When contact calls were not described in the Handbook of the Birds of the World Alive^69^ (hereafter HBW), we used the most common type of call recorded, avoiding alarm-related calls. By sampling one species per genus, we optimize taxonomic breadth and evenness, contributing to a representative sampling of species diversity in the Passerida (Fig. 1). Within each genus, we prioritized using the species with the most recordings available, to increase the likelihood of finding good quality recordings of both song and calls. While this is not a random choice of species within genera, using well-recorded species, which are likely common species or species easy to record, does not bias choice of species in a manner related to the hypotheses tested here.

We measured peak frequency (frequency with highest cumulative sound amplitude) in up to three recordings per species, and the within-species repeatability of these measurements^70^ was very high and identical for songs (R = 0.87) and calls (R = 0.86), indicating a similar accuracy when quantifying species differences in either songs or calls. The within-species repeatability for song duration was also high (R = 0.70), although not as high as for frequency, perhaps because birds can often terminate their songs at different points. In total we measured 3056 different recordings, on average 2.90 recordings of songs per species, and 2.29 recordings of calls per species. In the “Supporting information” (Appendix S1) we give detailed methods for obtaining and measuring acoustic recordings. The “Supporting information” (Appendix S1) also shows that further increasing the number of recordings per species does not increase within-species repeatability appreciably, neither for songs or calls, indicating that our sampling was sufficient to evaluate species differences in the sound frequency of songs and calls.

### Complete dataset and phylogenies

We took the body mass of each species from a data paper^71^. Also from this data paper, we quantified the proportion of time foraging in forested habitats as the sum of the proportion of time in all categories that unambiguously refer to forest: understory, mid-high strata, and canopy. Even though some habitat categories in the data paper^71^ do not unambiguously distinguish forest from non-forest, and the data refer to foraging habitat rather than specifically to the location of singing posts, these data nonetheless provide a better, and more nuanced evaluation of habitat differences between species than a dichotomous categorization of forest vs. non-forest (see also the discussion, for comparison with results using different proxies for habitat type).

Figure S3 in the “Supporting information” shows the distribution of the data across species, both acoustic, body mass and habitat. Peak frequencies (measured in a ratio scale^72^), log-transformed song duration, and body mass all had approximately bell-shaped distributions (Fig. S3A–E). The proportion of time foraging in forested habitat ranged from 0 to 1 among species, with species that forage more than 50% of the time in forests being only slightly more represented than those foraging less than 50% of the time in forests (“Supporting information”; Fig. S3F). The complete dataset is provided in the “Supporting information” (Tables S2, S3).

We used phylogenetic trees from Birdtree.org^73^, including all 591 species in the dataset. These phylogenies were constructed on the Hackett backbone^74^, using available molecular information in a Bayesian framework to obtain a large number of probable trees^73^. An example phylogenetic tree is shown in Fig. 1. We based all analyses on 1000 different phylogenetic trees to account for phylogenetic uncertainty (as recommended by^75^).

### Analyses

We ran all phylogenetic comparative analyses in R v. 3.5.1^76^, using the package caper (v 1.0.1^77^). First, we estimated the phylogenetic signal of each species trait (song peak frequency and duration, call peak frequency, body mass, and the proportion of time in forested habitat) using Pagel’s *λ*^32^, weight-averaged across the 1000 phylogenetic trees. We weighted results based on the Akaike’s Information Criterion (AIC) of each tree’s model output (following^78^). AIC-weighted averaging takes into account phylogenetic uncertainty and the parsimony of models from each tree^75,78^.

Separately for songs and for calls, we tested whether peak frequency was associated with body size and habitat type across species, using a phylogenetic generalized least squares (PGLS) regression model of peak frequency on body mass and on the proportion of time in forested habitat. The parameter *λ* was estimated in all regression models, to adjust the phylogenetic correction to the degree of phylogenetic signal in the model^79,80^. As before, we used AIC weights for model averaging of results over the 1000 trees, including *λ*, *β*, and standard errors. The weighted average of *β* (partial regression coefficient) was standardized (*β*_*st*_) by multiplying by the standard deviation of the corresponding predictor trait, and dividing by the standard deviation of the dependent trait (peak frequency). We ran a student’s t-test comparing the *β*_*st*_ of peak frequency on body mass between songs and calls (following^81^), and based on the AIC weight-averaged values of *β*_*st*_ and associated standard errors from the above multiple PGLS regressions models.

Finally, to test if longer, more elaborate songs, were associated with an increase or a decrease in sound frequency across species, we added song duration as a predictor to the above multiple PGLS regression model of peak song frequency on body mass and proportion of time in forested habitat. There appear to be no issues of multicollinearity in these multiple PGLS regression models, because the three predictors were not associated (PGLS of song duration on mass: *β*_*st*_ = 0.05, P = 0.31, model *λ* = 0.45; PGLS of song duration on habitat: *β*_*st*_ = 0.06, P = 0.17 model *λ* = 0.45; PGLS of mass on habitat: *β*_*st*_ = − 0.03, P = 0.39, model *λ* = 0.89; N = 591 spp. in all cases). To test if longer songs evolved associated with larger, non-directional deviations to the predicted optimum sound frequency for each species body size and habitat, we first computed residual frequency from the multiple PGLS regression model of song peak frequency on body mass and proportion of time in forested habitat; these residuals were weight-averaged across the 1000 trees, as explained before. We then ran a PGLS regression of the absolute values of these residual frequencies (large absolute values of residuals indicating a larger deviation to optimal sound frequency) on song duration. As above, PGLS model results were AIC weight-averaged across 1000 phylogenies.

## Supplementary Information

Supplementary Tables.

Supplementary Information.
