# Supplementary material for: Contingency and determinism in the evolution of bird song sound frequency
Source: Sci Rep. 2021 Jun 2;11:11600. doi: 10.1038/s41598-021-90775-6 (PMC8172888; doi:10.1038/s41598-021-90775-6)
Supplement: Supplementary file 2 — Supplementary Information. [file 41598_2021_90775_MOESM2_ESM.pdf]

Supplementary appendix and figures for: *Contingency and determinism in the evolution of bird song sound frequency*

Jakob Isager Friis, Torben Dabelsteen, Gonçalo C. Cardoso

Contents

Supplementary Appendix

Appendix S1: In depth description on the gathering and measuring of acoustic data, and repeatability estimations

Supplementary Figures

Figure S1. Example spectrograms of songs and calls

Figure S2. Repeatability of acoustic measurements

Figure S3. Histograms of the data for all species

References

Appendix S1

We measured peak sound frequency (hereafter, peak frequency) of songs and calls in Passerida species, as well as duration of songs, using acoustic recordings from the citizen-science online database *Xenocanto* (www.xeno-canto.org; hereafter XC). Following the phylogeny presented by Johansson, Fjeldså and Bowie[1], with modifications for Paroidea[2] and *Regulus*[3], we include in the parvorder Passerida the superfamilies Muscicapoidea, Sylvioidea and Passeroidea, as well as families Picathartidae, Eupetidae, Chaetopidae, Petroicidae, Hyliotidae, Regulidae, Elachuridae, Pnoepygidae, Bernieridae, Timaliidae and Pellorneidae. The clade comprises a total of 76 families, as used in the Handbook of the Birds of the World Alive[4] (hereafter HBW), and correspond to all Passeriform families after Picathartidae in the phylogenetically ordered list of HBW. The list of these 76 families and their genera is in Table S1, and figures in the main text colour species belonging to the 4 largest superfamilies[(following 1–3)].

We first measured recordings for only one species per family to evaluate how many different sound recordings per species would be necessary to robustly evaluate species differences in acoustic traits. Afterwards, we expanded the dataset to one species per genus. For the dataset consisting of one species per family, we selected 5 recordings of songs and 5 recordings of calls per species, choosing recordings informed by the descriptions of song and calls in the full text "voice" entry of HBW. For each family, we searched the XC online database for the genus with the most recordings, and then the species in that genus with the most recordings. If that species had less than 5 good quality recordings of songs or less than 5 good quality recordings of calls, then we chose the next most recorded species, until finding a usable species for the family. If no species in the family had a sufficient number of good quality recordings, then the family was not included. Species without a clear distinction between songs and calls, as described in HBW, or with no clearly identifiable advertisement song (e.g., Aegithalidae[4]) were also not included. Selecting recordings of calls sought to avoid those described as alarm calls and anxiety calls, and prioritised using contact calls when they were described as such in HBW. We also prioritised using recordings of songs and calls with little background noise, no overlapping transient noise, and also from recordings from different times and locations. Species that only had recordings of antiphonal singing (duet singing) were not used if pairs could not be separated. Species that only had recordings of chorus songs were also not included. In total, we could find species with 5 good quality recordings of songs and calls for 48 out of the 76 Passerida families.

We downloaded audio files and converted them to Wave format with a sample rate of 44.1 kHz, using the warbleR package[5] in R v. 3.5.1[6] or the audio converter fre:ac (www.freac.org). We then analysed each recording with the software Avisoft-SasLab Pro (Avisoft Bioacoustics, Berlin, Germany), to measure acoustic parameters for each song or call, as follows. We chose up to 5 songs or 5 calls from each recording for measurements, based on sound quality within the recording. We then identified individual songs and calls based on their descriptions in HBW and measured their duration (in seconds, to the 3rd decimal place) by marking each song or call from its onset to its end, excluding any trailing reverberation or echoes, using a label in the spectrogram view of Avisoft (examples in Fig. S1). Spectrograms used FFT length 512, flat top window, and 50% window overlap, which correspond to a time resolution of 5.8

ms and a frequency resolution of 86 Hz. When HBW did not contain a detailed description of song, we identified individual songs as segments of continuous singing separated from other songs by more than one second. For continuous singers, which can have very long and uninterrupted songs, we trimmed songs at the maximum duration of 60 seconds for measurements; very few song measurements were this long (only 1% of song measurements >59sec., comprising a total of 32 out of 591 species). In cases where high-amplitude songs are interspersed amidst separate units of noticeably lower amplitude short-range singing, we marked the long-range part for measurement. We applied a high-pass filter of 1 kHz to discard low-frequency noise, as most Passerida song and contact calls are above this range, and then used the automatic measurement tool in Avisoft to measure peak sound frequency for each previously marked song or call label. Peak frequency is the frequency with maximum amplitude in the power spectrum of the entire song or call. We checked all frequency measurements visually on spectrograms. If noise affected those measurements (i.e., measured frequency fell on the 1 kHz high-pass or on extraneous bursts of noise) the affected acoustic parameters were removed from the data, unless the issue was due to a discrete burst of noise, not overlapping in frequency with the bird vocalization, that could be deleted to fix the measurement. Frequency measurements, in Hz, were $\log_{10}$-transformed before averaging, because logarithmic (i.e., ratio) scales of sound frequency conform to how animals perceive and modulate sound frequency, and is therefore more appropriate for comparative analyses[7]. The distribution of song durations was strongly right-skewed, and therefore we also $\log_{10}$-transformed mean song duration per species.

Fig. S2 shows the within-species repeatability of acoustic measurements, using all 5 recordings measured per species, or using only a smaller subset of recordings per species. In all cases, repeatability was computed using mean measurements per recording and running a linear mixed effect model based estimation in the package rptR[8], according to recommendations for Gaussian data[9]. To compute repeatability using fewer than 5 recordings per species, we wrote R code to randomly select 2, 3 or 4 recordings per species and compute within-species repeatability with those subsets of data; we repeated this sub-sampling procedure 100 times. Fig. S2 shows that the medians of the repeatability estimates were consistently high, in all cases > 0.8, and they did not decrease appreciably for small sample sizes (even for only 2 recordings per species; Fig. S2). The lowest estimate across all instances of random sampling was high

too, almost never falling below 0.70 (Fig. S2). These high repeatability estimates indicate that the variation among recordings of the same species is small compared to among-species variation, and thus, even few recordings per species allow reliable cross-species comparisons of these acoustic traits. We therefore decided to sample up to 3 recordings of songs and of calls per species, for the remaining species.

For the complete dataset, consisting of one species per genus, we searched for up to 3 recordings of songs and up to 3 recordings of calls per species in XC, and used the same species selection procedure described above in order to select one species per genus. Briefly, we started with the species with the most recordings in each genus and, if the number of good quality recordings of song and calls were not sufficient, we moved to the next most recorded species; genera without a sufficiently recorded species were not included. Also as before, we avoided species without a clear distinction between songs and calls, avoided alarm and anxiety calls and prioritised using contact calls. For some species good quality recordings were available for song but not for calls, resulting in a larger number of species with song measurements (591 spp.) than with call measurements (505 spp.). In the remaining analyses, all acoustic measurements were averaged per species, resulting in a single data point per species for each type of acoustic measure.

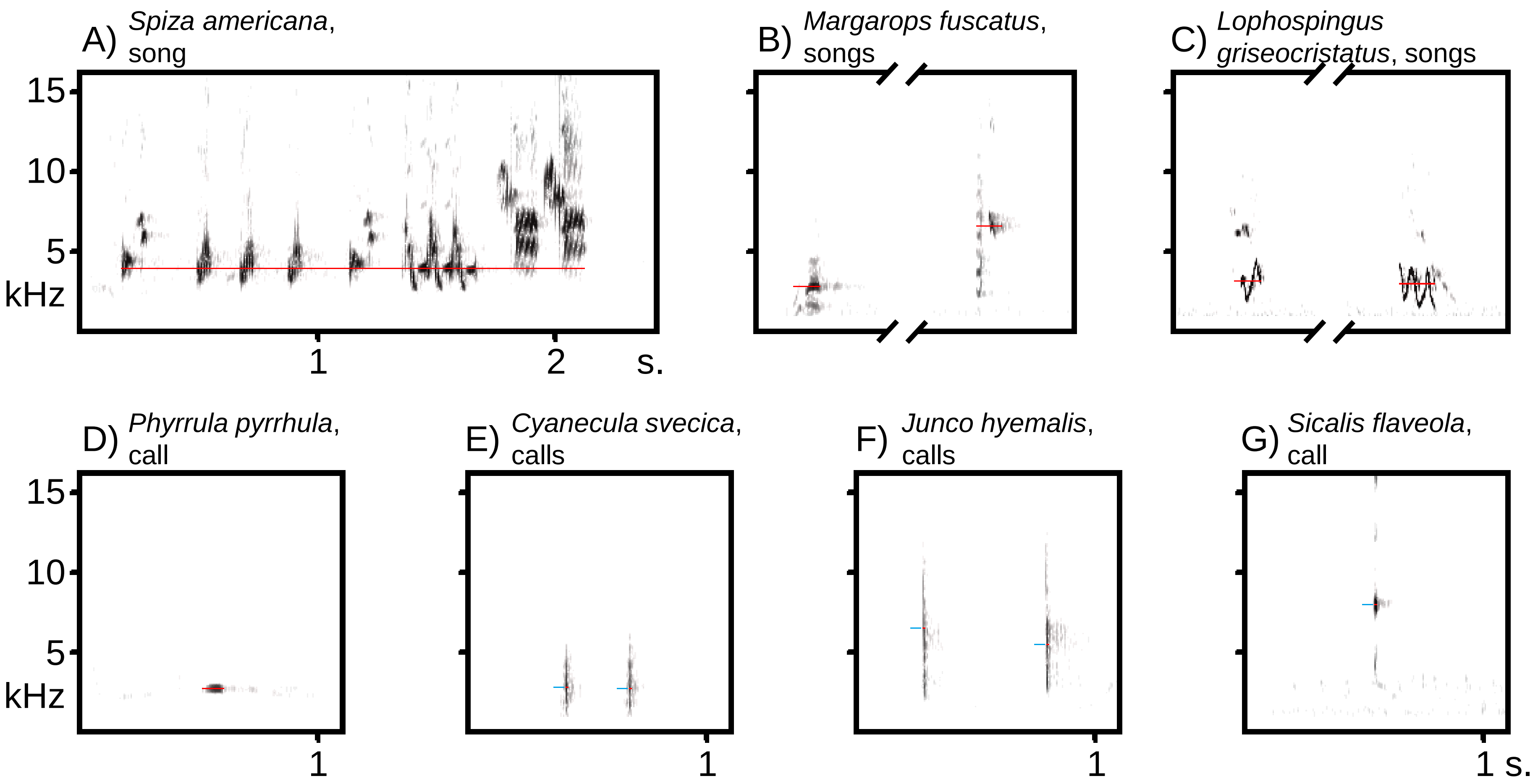

Figure S1. Examples of songs (A-C) and calls (D-G) analysed in this work. Shown are examples of a song (A) and a call (D) with durations close to the median duration across species, and of songs (B, C) and calls (E-G) with the shortest durations among all our measurements. Red lines indicate peak frequency, as measured and plotted automatically in Avisoft (for ease of viewing, a blue line was added before the red mark of the short calls). Spectrogram settings are as described in the methods, except that here, for ease of viewing, spectrograms are trimmed above 15 kHz. The time resolution on spectrograms (i.e., the duration of a pixel) is 5.8 ms. Note that we marked durations excluding trailing reverberation or echoes and, since Avisoft measures duration as the onset-to-onset distance between pixel markings, very short calls marked as two-pixels-long (as in E-G) are automatically measured as 5 rather than 11 ms.

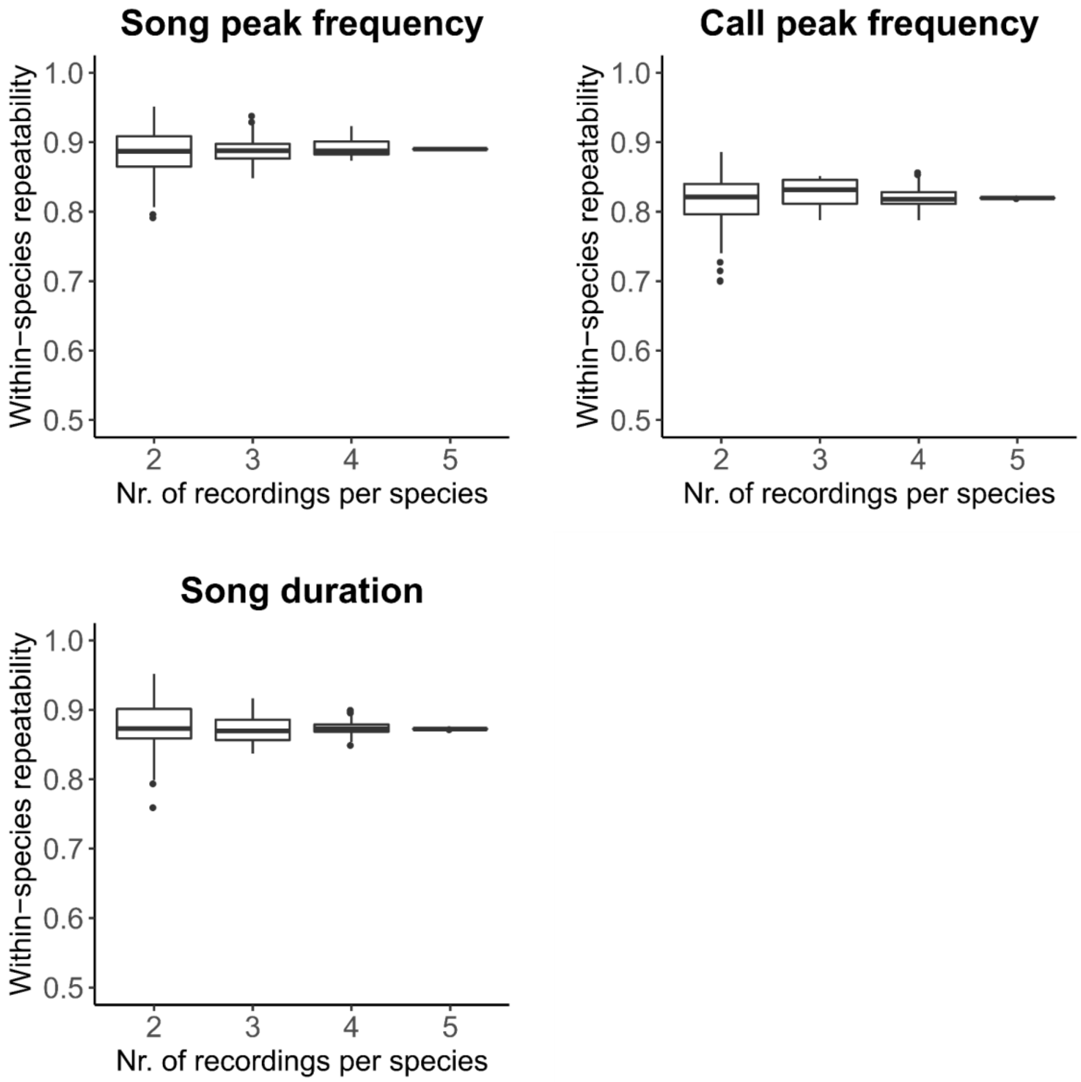

Figure S2. Repeatability of acoustic measurements for a subset of 48 species from 48 families. Within-species repeatability of acoustic measurements across recordings, using sample sizes of 1 to 5 recordings per species. Boxplots indicate median values (central band), 25th and 75th quartiles (boxes), range excluding outliers (lines), and outliers (dots).

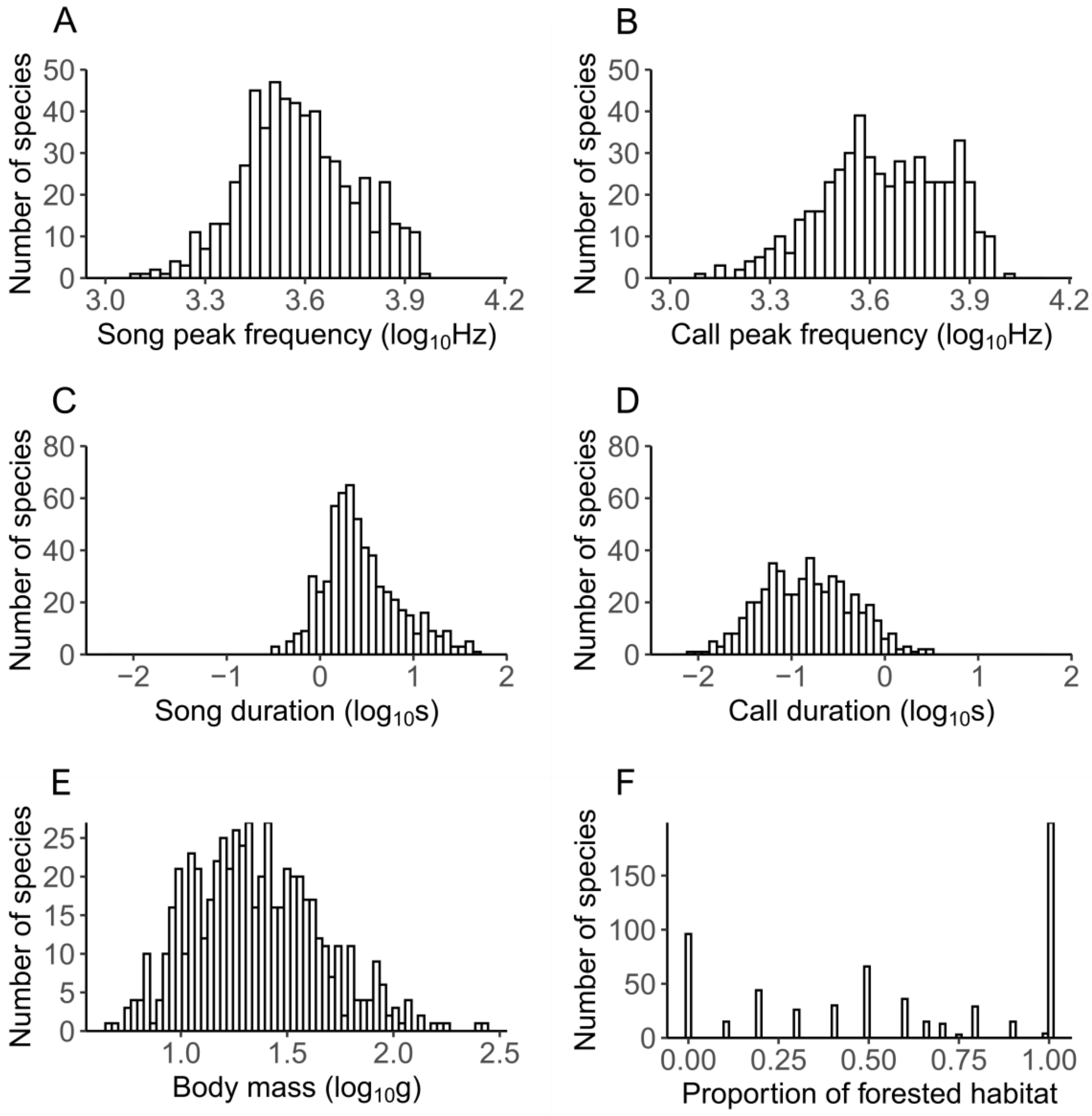

Figure S3. Histograms of species data. Histograms of the peak frequency of (A) songs and (B) calls, duration of songs (C) and calls (D), as well as measures of (E) body mass and (F) the proportion of forested habitat.

References

1. Johansson, U. S., Fjeldså, J. & Bowie, R. C. K. Phylogenetic relationships within Passerida (Aves: Passeriformes): A review and a new molecular phylogeny based on three nuclear intron markers. *Mol. Phylogenet. Evol.* **48**, 858–876 (2008).

2. Sangster, G. *et al.* Taxonomic recommendations for Western Palaearctic birds:

10th report. *Ibis (Lond. 1859).* **157**, 193–200 (2015).

3. Wu, L. *et al.* A phylogeny of the Passerida (Aves: Passeriformes) based on mitochondrial 12S ribosomal RNA gene. *Avian Res.* **6**, 4–11 (2015).
4. del Hoyo, J., Elliott, A., Christie, D. . & de Juana, E. HBW Alive: Handbook of the Birds of the World Alive. *Lynx edition, Barcelona* (2018). Available at: https://www.hbw.com/. (Accessed: 23rd September 2018)
5. Araya-Salas, M. & Smith-Vidaurre, G. warbleR: an r package to streamline analysis of animal acoustic signals. *Methods Ecol. Evol.* **8**, 184–191 (2017).
6. R Team. R: A language and environment for statistical computing. R Foundation for Statistical Computing, Vienna, Austria. (2014).
7. Cardoso, G. C. Using frequency ratios to study vocal communication. *Anim. Behav.* **85**, 1529–1532 (2013).
8. Stoffel, M. A., Nakagawa, S. & Schielzeth, H. rptR: repeatability estimation and variance decomposition by generalized linear mixed-effects models. *Methods Ecol. Evol.* **8**, 1639–1644 (2017).
9. Nakagawa, S. & Schielzeth, H. Repeatability for Gaussian and non-Gaussian data: A practical guide for biologists. *Biol. Rev.* **85**, 935–956 (2010).
